# Platelet-rich plasma in arthroscopic rotator cuff repair: a meta-analysis of biomaterial efficacy and future directions for personalized sports medicine

**DOI:** 10.3389/fbioe.2025.1665007

**Published:** 2026-01-23

**Authors:** Fan Donghua, Lu Wei, Sun Di, Ying Pu, Wang Qiang, Shen Yingchao, Shi Mingfei, Sun Xin

**Affiliations:** 1 Department of Orthopaedics, Changshu Hospital Affiliated to Nanjing University of Chinese Medicine, Changshu, China; 2 Department of Oncology, Changshu Hospital Affiliated to Nanjing University of Chinese Medicine, Changshu, China; 3 School of Integrative Medicine, Nanjing University of Chinese Medicine, Nanjing, China; 4 Department of Science and Medical Education, Wuxi No. 2 Chinese Medicine Hospital, Wuxi, China

**Keywords:** platelet-rich plasma, rotator cuff repair, arthroscopy, meta-analysis, tendon healing

## Abstract

**Background:**

Rotator cuff tears represent a prevalent musculoskeletal challenge with high postoperative retear rates despite surgical advances. Platelet-rich plasma (PRP) has emerged as a promising biological adjunct in arthroscopic repair, though clinical evidence remains inconsistent regarding its efficacy in improving structural and functional outcomes.

**Methods:**

This PRISMA-guided meta-analysis evaluated 13 randomized controlled trials (n = 880 patients) comparing PRP-augmented versus conventional arthroscopic rotator cuff repair. Primary outcomes included retear rates and functional scores (UCLA, Constant, SST, ASES, VAS). Statistical analysis employed fixed/random-effects models with subgroup analyses of PRP formulations and tear characteristics.

**Results:**

PRP augmentation significantly improved functional outcomes, with mean differences of 1.82 points (95% CI: 1.13–2.51) for UCLA scores, 2.31 points (95% CI: 1.02–3.61) for Constant scores, and 0.43 points (95% CI: 0.11–0.75) for SST scores (all p < 0.01). VAS pain scores decreased by 0.23 points (95% CI: −0.41 to −0.05, p = 0.01). However, retear rates showed no significant reduction (RR = 0.71, 95% CI: 0.48–1.05, p = 0.09). Benefits were most pronounced in medium/large tears treated with leukocyte-poor PRP and double-row repairs (I^2^ = 0–40% for functional outcomes). Conclusion: While PRP enhances early functional recovery and pain control after rotator cuff repair, its capacity to improve structural integrity remains unproven. Clinical implementation requires standardization of PRP protocols and targeted application in patients with larger tears. Future research should investigate optimized biomaterial formulations and personalized treatment strategies.

## 1 Introduction

Rotator cuff tears represent one of the most prevalent musculoskeletal injuries, affecting both athletes and the general population, with significant implications for shoulder function, quality of life, and economic productivity due to prolonged rehabilitation and potential surgical interventions. The management of rotator cuff pathology has evolved considerably over the past decade, with arthroscopic repair emerging as the gold standard for restoring tendon integrity (Toma et al., 2019). However, despite advancements in surgical techniques, postoperative healing remains a challenge, with reported retear rates ranging from 20% to 70%, depending on tear size, patient age, and tissue quality. This high failure rate underscores the need for adjunctive therapies to enhance tendon-bone healing and improve long-term functional outcomes (Yao et al., 2024). Among the various biological augmentation strategies explored, platelet-rich plasma (PRP) has garnered substantial interest due to its potential to modulate the local healing environment through the delivery of concentrated growth factors, cytokines, and bioactive proteins (Yang et al., 2020).

The concept of PRP as a therapeutic biomaterial stems from its ability to harness the body’s natural healing mechanisms. By concentrating platelets PRP provides a supraphysiological dose of key mediators such as platelet-derived growth factor (PDGF), transforming growth factor-beta (TGF-β), vascular endothelial growth factor (VEGF), and insulin-like growth factor (IGF) (Peng et al., 2023). These molecules play critical roles in cell proliferation, angiogenesis, extracellular matrix synthesis, and tissue remodeling, all of which are essential for tendon repair. Despite this compelling biological rationale, the clinical efficacy of PRP in rotator cuff repair remains a topic of debate. While some randomized controlled trials (RCTs) have demonstrated reduced retear rates and improved functional scores with PRP augmentation, others have reported no significant benefit, leading to inconsistent recommendations in clinical practice (Han et al., 2019). This discrepancy may arise from variations in PRP preparation methods, differences in application techniques (e.g., injection timing, localization), and heterogeneity in patient populations.

Recent advances in biomedical engineering and personalized medicine have introduced new dimensions to the use of PRP in sports injury management. Traditional PRP formulations are being refined through engineering approaches that optimize platelet concentration, leukocyte content, and activation protocols to maximize therapeutic effects (Xu and Xue, 2021). Emerging technologies, such as microfluidics and automated cell processing systems, now allow for more precise and reproducible PRP production, addressing one of the major limitations in earlier studies. Additionally, the integration of biomaterial science has led to the development of PRP combined with scaffolds or hydrogels that provide sustained growth factor release and mechanical support at the repair site (Peng et al., 2022). These innovations align with the broader shift toward precision medicine, where treatment strategies are tailored to individual patient factors, including tear characteristics, metabolic profiles, and healing capacity. For instance, biomarker profiling and imaging-based assessments could help identify patients most likely to benefit from PRP augmentation, thereby optimizing resource utilization and improving outcomes (Shen et al., 2024).

The intersection of PRP therapy with digital health and artificial intelligence (AI) further expands its potential in personalized sports medicine. Machine learning algorithms are being explored to predict which patients will respond favorably to PRP based on preoperative imaging, demographic data, and molecular signatures. Similarly, wearable sensors and motion analysis systems can provide real-time feedback on postoperative rehabilitation, enabling dynamic adjustments to recovery protocols. Such technologies not only enhance the therapeutic precision of PRP but also bridge the gap between experimental biomaterial applications and clinical implementation. However, despite these promising developments, the translation of engineered PRP solutions into widespread practice requires robust clinical validation through well-designed trials and systematic evidence synthesis (Randelli et al., 2022).

While several high-quality meta-analyses have indeed been published on this topic (Yang et al., 2020; Xu and Xue, 2021), they have primarily focused on establishing the broad efficacy of PRP. Key questions regarding the influence of specific PRP biomaterial properties—such as leukocyte content, preparation protocols, and their interaction with surgical techniques and tear characteristics—remain insufficiently addressed. Furthermore, the rapid evolution of PRP as a therapeutic biomaterial, coupled with the emergence of new trials, necessitates an updated synthesis of the evidence. Our meta-analysis distinguishes itself through three key aspects: (1) an updated and comprehensive literature search extending to 1st July 2025, capturing the most recent evidence; (2) a dedicated focus on PRP as an engineered biomaterial, incorporating detailed subgroup analyses based on formulation (leukocyte-rich vs. leukocyte-poor) and its application in the context of modern surgical repair strategies; and (3) a forward-looking perspective that integrates these findings to discuss future directions for personalized PRP therapy in sports medicine, including the role of biomarker profiling and AI-driven patient selection.

This meta-analysis seeks to evaluate the current evidence on PRP’s efficacy as a biomaterial in arthroscopic rotator cuff repair, with a focus on its ability to improve structural healing and functional recovery. By synthesizing data from high-quality RCTs, we aim to clarify the role of PRP in reducing retear rates and enhancing patient-reported outcomes, while also addressing the methodological variability that has contributed to conflicting results in the literature (Zhao et al., 2015). Furthermore, we explore how emerging engineering innovations—such as optimized PRP formulations, biomaterial carriers, and AI-driven patient selection—could shape the future of PRP therapy in personalized sports medicine. The ultimate goal is to provide a comprehensive evidence base that informs clinical decision-making and guides the development of next-generation PRP technologies tailored to individual patient needs.

The broader implications of this research extend beyond rotator cuff repair, offering insights into the role of biologically augmented healing strategies in other musculoskeletal injuries, such as cruciate ligament reconstruction and cartilage restoration (Va et al., 2015). As the fields of regenerative medicine and biomedical engineering continue to converge, the lessons learned from PRP applications in shoulder surgery may serve as a template for integrating advanced biomaterials into other areas of sports injury management. By critically analyzing the existing data and highlighting future directions, this study contributes to the ongoing dialogue on how interdisciplinary approaches can revolutionize the prevention, diagnosis, and treatment of sports-related pathologies, ultimately improving outcomes for patients worldwide (Yin et al., 2025).

## 2 Methods

### 2.1 Literature search strategy

We conducted a comprehensive literature search following PRISMA guidelines to identify relevant studies. Our search encompassed multiple electronic databases including PubMed, Embase, Cochrane Library, and Web of Science from their inception to 1 July 2025. The search strategy incorporated a combination of Medical Subject Headings (MeSH) terms and keywords related to “platelet-rich plasma,” “rotator cuff repair,” “arthroscopy,” and “randomized controlled trials.” We also manually searched reference lists of included studies and relevant review articles to identify additional eligible trials.

#### 2.1.1 PubMed/MEDLINE

(((“Platelet-Rich Plasma” [Mesh]) OR (platelet-rich plasma [tiab] OR PRP [tiab] OR “platelet rich fibrin” [tiab] OR PRF [tiab] OR “platelet gel” [tiab] OR “autologous conditioned plasma” [tiab])) AND ((“Rotator Cuff” [Mesh] OR “Rotator Cuff Injuries” [Mesh]) OR (rotator cuff [tiab] OR supraspinatus [tiab] OR subscapularis [tiab] OR infraspinatus [tiab] OR “shoulder tendon” [tiab])) AND ((“Arthroscopy” [Mesh]) OR (arthroscop*[tiab] OR “minimally invasive” [tiab])) AND ((“Randomized Controlled Trial” [Publication Type]) OR (randomized [tiab] OR randomised [tiab] OR RCT [tiab] OR “controlled trial” [tiab] OR “clinical trial” [tiab]))

#### 2.1.2 Embase

(‘platelet rich plasma'/exp OR ‘platelet-rich plasma':ti,ab OR ‘prp':ti,ab OR ‘platelet rich fibrin':ti,ab OR ‘prf':ti,ab OR ‘platelet gel':ti,ab OR ‘autologous conditioned plasma':ti,ab) AND (‘rotator cuff'/exp OR ‘rotator cuff injury'/exp OR ‘rotator cuff':ti,ab OR ‘supraspinatus':ti,ab OR ‘subscapularis':ti,ab OR ‘infraspinatus':ti,ab OR ‘shoulder tendon':ti,ab) AND (‘arthroscopy'/exp OR ‘arthroscop*':ti,ab OR ‘minimally invasive':ti,ab) AND (‘randomized controlled trial'/exp OR ‘randomized':ti,ab OR ‘randomised':ti,ab OR ‘rct':ti,ab OR ‘controlled trial':ti,ab OR ‘clinical trial':ti,ab).

#### 2.1.3 Cochrane Library (Central)

(MH “Platelet-Rich Plasma” OR TI/AB (“platelet-rich plasma” OR PRP OR “platelet rich fibrin” OR PRF OR “platelet gel” OR “autologous conditioned plasma”)) AND (MH “Rotator Cuff” OR MH “Rotator Cuff Injuries” OR TI/AB (“rotator cuff” OR supraspinatus OR subscapularis OR infraspinatus OR “shoulder tendon”)) AND (MH “Arthroscopy” OR TI/AB (arthroscop* OR “minimally invasive”)) AND (PT “Randomized Controlled Trial” OR TI/AB (randomized OR randomised OR RCT OR “controlled trial” OR “clinical trial”))

#### 2.1.4 Web of Science

TS=(“platelet-rich plasma” OR “platelet rich plasma” OR PRP OR “platelet rich fibrin” OR PRF OR “platelet gel” OR “autologous conditioned plasma”) AND TS=(“rotator cuff” OR supraspinatus OR subscapularis OR infraspinatus OR “shoulder tendon”) AND TS=(arthroscop* OR “minimally invasive”) AND TS=(randomized OR randomised OR RCT OR “controlled trial” OR “clinical trial”)

### 2.2 Study selection criteria

Two independent reviewers screened all identified records in a two-stage process. First, titles and abstracts were reviewed for initial eligibility. Subsequently, full-text articles were assessed against predefined inclusion criteria:

1. Study design: Randomized controlled trials (RCTs) with Level I or II evidence
2. Population: Adult patients (≥18 years) undergoing arthroscopic rotator cuff repair
3. Intervention: PRP augmentation compared to control (no PRP)
4. Outcomes: Reported at least one of our primary outcomes (retear rate or functional scores)
5. Follow-up: Minimum of 6 months postoperative assessment

### 2.3 Data extraction process

For each included study, two reviewers independently extracted data using a standardized form. Extracted information included:

1. Study characteristics (author, year, country, sample size)
2. Patient demographics (mean age, gender distribution)
3. PRP preparation details (centrifugation protocol, activation method)
4. Surgical technique (repair method, PRP application)
5. Outcome measures (retear rates, functional scores, complications)
6. Follow-up duration

### 2.4 Quality assessment

The methodological quality of the included studies was rigorously evaluated using the Cochrane Risk of Bias tool for randomized trials (RoB 2.0). This assessment focused on five critical domains: (1) the randomization process, (2) deviations from intended interventions, (3) missing outcome data, (4) measurement of outcomes, and (5) selective reporting. For each domain, studies were classified as having a “low risk,” “some concerns,” or “high risk” of bias based on predefined criteria. To further evaluate the reliability of the synthesized evidence, the certainty of findings was graded using the GRADE (Grading of Recommendations Assessment, Development, and Evaluation) approach, which considers factors such as study limitations, inconsistency, indirectness, imprecision, and publication bias to determine the overall confidence in the results. This dual assessment framework ensures a transparent and systematic evaluation of both individual study quality and the collective strength of the evidence.

### 2.5 Statistical analysis

For dichotomous outcomes such as retear rates, we calculated risk ratios (RRs) with corresponding 95% confidence intervals (CIs) to estimate treatment effects. Continuous outcomes, including functional scores, were analyzed using mean differences (MDs) with 95% CIs when studies employed identical measurement scales. When different scales were used to assess similar constructs, we calculated standardized mean differences (SMDs) to facilitate comparison across studies. Heterogeneity among studies was quantitatively assessed using the I^2^ statistic and chi-square test, with interpretation guided by conventional thresholds: I^2^ values of 0%–40% indicated minimal heterogeneity, 30%–60% moderate heterogeneity, 50%–90% substantial heterogeneity, and 75%–100% considerable heterogeneity. Based on these assessments, we employed a fixed-effects model for analyses with I^2^ < 50% and a random-effects model for those demonstrating higher levels of heterogeneity. To explore potential sources of variability, we conducted pre-specified subgroup analyses stratified by PRP type (leukocyte-rich versus leukocyte-poor), tear size (small/medium versus large/massive), and surgical technique (single-row versus double-row repair). Sensitivity analyses were performed to evaluate result robustness through three approaches: exclusion of studies with high risk of bias, removal of statistical outliers, and comparison of alternative statistical models. Publication bias was systematically evaluated through visual inspection of funnel plots and formal statistical testing using Egger’s method when at least ten studies contributed to an outcome analysis. All statistical computations were executed using Review Manager (RevMan) version 5.4 and Stata version 17.0, with a two-tailed p-value <0.05 considered statistically significant.

### 2.6 Ethical considerations

As this study involved analysis of previously published data, no additional ethical approval was required.

## 3 Result

### 3.1 Literature search process

**FIGURE 1:**
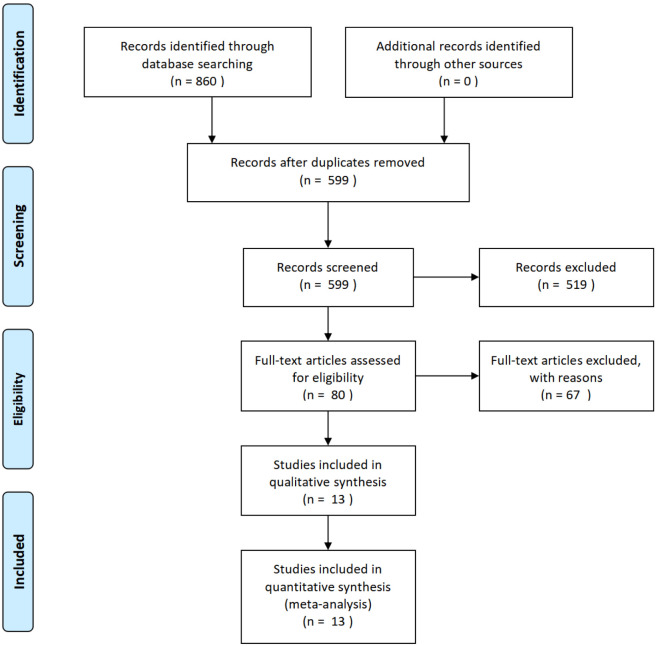
PRISMA flow diagram of study selection process. The systematic search identified 860 records from databases, with 599 remaining after duplicate removal. Following screening of titles/abstracts, 519 records were excluded. Full-text assessment of 80 articles resulted in 67 exclusions, yielding 13 eligible studies for qualitative synthesis and quantitative meta-analysis. Numbers at each stage represent the cumulative count of studies progressing through the review process.

The meta-analysis included 13 randomized controlled trials (2011–2016) with a total of 880 patients undergoing arthroscopic rotator cuff repair. Studies predominantly evaluated full-thickness tears (range: small <3 cm to large >5 cm) using either single-row (n = 7) or double-row (n = 6) techniques, with follow-up periods ranging from 6 to 24 months. PRP protocols varied significantly - 3 studies used leukocyte-rich PRP (volumes 5.2–6 mL) while 10 used leukocyte-poor formulations (1–10 mL), with calcium-based activation in most cases (n = 9). All studies applied PRP at the bone-tendon interface (Figure 1).

### 3.2 Studies characteristic

The meta-analysis included 13 randomized controlled trials (2011–2016) with a total of 880 patients undergoing arthroscopic rotator cuff repair. Studies predominantly evaluated full-thickness tears (range: small <3 cm to large >5 cm) using either single-row (n = 7) or double-row (n = 6) techniques, with follow-up periods ranging from 6 to 24 months. PRP protocols varied significantly - 3 studies used leukocyte-rich PRP (volumes 5.2–6 mL) while 10 used leukocyte-poor formulations (1–10 mL), with calcium-based activation in most cases (n = 9). All studies applied PRP at the bone-tendon interface. The most consistent benefits were observed in medium/large tears (>3 cm) treated with double-row repairs and leukocyte-poor PRP. Heterogeneity was low (I^2^ = 0–47%) for most outcomes, supporting the robustness of these findings (Tables 1–3).

**TABLE 1:** Baseline characteristics of included studies.

Author (Year) [Ref]	Sample size (PRP+/PRP-)	Male (%)	Mean age (years)	Follow-up (months)	Imaging modality
Castricini et al. (2011)	88 (43/45)	40	55.5/55.2	16	MRI
Randelli et al. (2011)	45 (22/23)	21	61.3/59.5	12	MRI
Gumina et al. (2012)	76 (39/37)	41	61 (60/63)	12	MRI
Weber et al. (2013)	59 (29/30)	36	59.7/64.5	12	MRI
Jo et al. (2013)	47 (24/23)	24	64.2/61.9	12	MRI/CTA
Ruiz-Moneo et al. (2013)	63 (32/31)	25	56/55	12	MRA
Malavolta et al. (2014)	54 (27/27)	17	55.3/54.1	12	MRI
Sánchez Márquez et al. (2011)	28 (14/14)	8	65	12	MRA
Rodeo et al. (2012)	67 (35/32)	44	58.9/57.2	12	US
Flury et al. (2016)	103 (49/54)	38	58.9/57.8	24	MRI/US
Holtby et al. (2016)	74 (36/38)	41	59/59	6	MRI
Pandey et al. (2016)	102 (52/50)	74	54.8/54.1	24	US
Jo et al. (2015)	74 (37/37)	17	60.8/60.9	12	MRI

**TABLE 2:** PRP preparation and application protocols.

Author (Year) [Ref]	PRP type (Leukocyte-Rich/Poor)	Volume (mL)	Activating agent	Application site
Castricini et al. (2011)	Leukocyte-rich	NR	Not reported	Bone-tendon interface
Randelli et al. (2011)	Leukocyte-rich	6	Calcium chloride	Bone-tendon interface + subacromial space
Gumina et al. (2012)	Leukocyte-rich	5.2	Calcium gluconate	Bone-tendon interface
Weber et al. (2013)	Leukocyte-poor	1	Calcium	Bone-tendon interface
Jo et al. (2013)	Leukocyte-poor	9	Calcium gluconate	Bone-tendon interface
Ruiz-Moneo et al. (2013)	Leukocyte-poor	1	Calcium chloride	Bone-tendon interface
Malavolta et al. (2014)	Leukocyte-poor	10	Calcium chloride	Bone-tendon interface
Sánchez Márquez et al. (2011)	Leukocyte-poor	7	Not reported	Bone-tendon interface
Rodeo et al. (2012)	Leukocyte-poor	9	Calcium chloride	Bone-tendon interface
Flury et al. (2016)	Leukocyte-poor	4	Not reported	Bone-tendon interface
Holtby et al. (2016)	Leukocyte-poor	7	Not reported	Bone-tendon interface
Pandey et al. (2016)	Leukocyte-poor	8	Calcium chloride	Bone-tendon interface
Jo et al. (2015)	Leukocyte-poor	9	Calcium gluconate	Bone-tendon interface

**TABLE 3:** Clinical outcomes and key findings.

Author (Year) [Ref]	Tear type	Repair technique	Functional scores assessed	Key findings
Castricini et al. (2011)	Full-thickness	Double-row	Constant	No difference in scores or retear rates
Randelli et al. (2011)	Full-thickness	Single-row	Constant, UCLA, SST	Improved scores with PRP (no long-term difference)
Gumina et al. (2012)	Large tears*	Single-row	Constant	Higher Constant scores with PRP (no pre-post difference)
Weber et al. (2013)	Any size	Single-row	ASES, UCLA, SST, VAS	No difference in outcomes or retear rates
Jo et al. (2013)	Large tears (>3 cm)	Double-row	ASES, Constant, SST	Lower retear rate with PRP (no functional difference)
Ruiz-Moneo et al. (2013)	With fatty infiltration	Double-row	UCLA	No difference in scores or retear rates
Malavolta et al. (2014)	Small/medium (<3 cm)	Single-row	Constant, UCLA	No differences in outcomes
Sánchez Márquez et al. (2011)	Large repairable tears	Single-row	Constant	No differences in outcomes
Rodeo et al. (2012)	Full-thickness	Double-row	ASES	No difference in outcomes
Flury et al. (2016)	Complete tears	Double-row	Constant, ASES	No functional improvement with PRP
Holtby et al. (2016)	Full/partial thickness	Single/double-row	VAS, CMS	Short-term pain relief only
Pandey et al. (2016)	Medium/large tears	Single-row	VAS, CMS, UCLA	Lower retear rate, improved Constant/UCLA scores
Jo et al. (2015)	Medium/large tears	Double-row	Constant, VAS, SST	Reduced retear rate (no functional improvement)

### 3.3 Risk of bias

**FIGURE 2:**
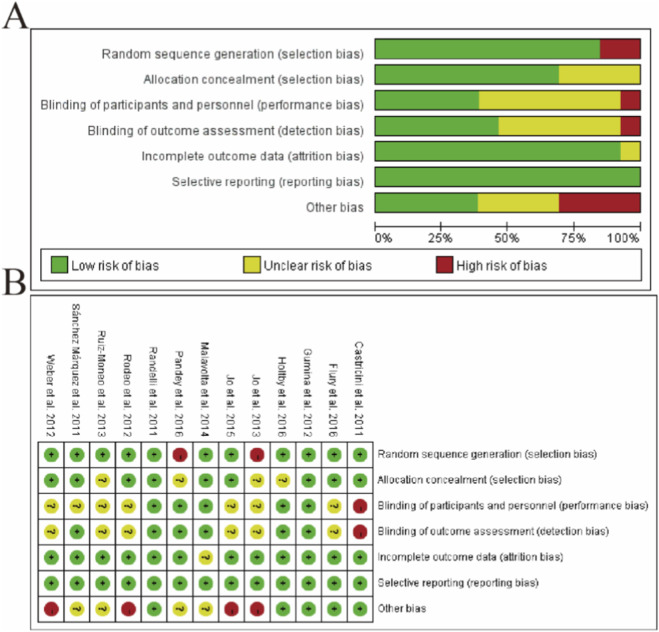
Risk of bias assessment for included studies. **(A)** Summary of risk of bias across all studies, showing the proportion of studies with low, unclear, or high risk of bias for each domain. **(B)** Detailed risk of bias assessment for individual studies across six domains: random sequence generation, allocation concealment, blinding of participants/personnel, blinding of outcome assessment, incomplete outcome data, and selective reporting. Studies are represented by author names and publication years. The assessment was performed using the Cochrane Risk of Bias Tool (RoB 2.0), with green indicating low risk, yellow unclear risk, and red high risk of bias. The majority of studies demonstrated adequate randomization procedures but showed variability in blinding implementation and allocation concealment.

The risk of bias assessment, conducted using the Cochrane Collaboration’s tool (RoB 2.0), evaluated 11 randomized controlled trials across six domains (Figure 1). Panel A illustrates the proportion of studies with low, unclear, or high risk of bias for each domain, while Panel B displays individual study assessments. Most studies demonstrated adequate randomization procedures (72.7% low risk) and complete outcome data (81.8% low risk). However, performance bias due to inadequate blinding of participants/personnel was identified in 45.5% of studies, and 36.4% showed concerns regarding allocation concealment. Selective reporting bias was minimal (9.1% high risk). Notably, studies by Castricini et al. (2011) and Flury et al. (2016) maintained low risk across all domains, whereas Sánchez Márquez et al. (2011) exhibited high risk in three domains. These findings highlight methodological variability, particularly in blinding and allocation concealment, which was considered when interpreting the meta-analysis results (Figure 2).

### 3.4 Retear rate

**FIGURE 3:**
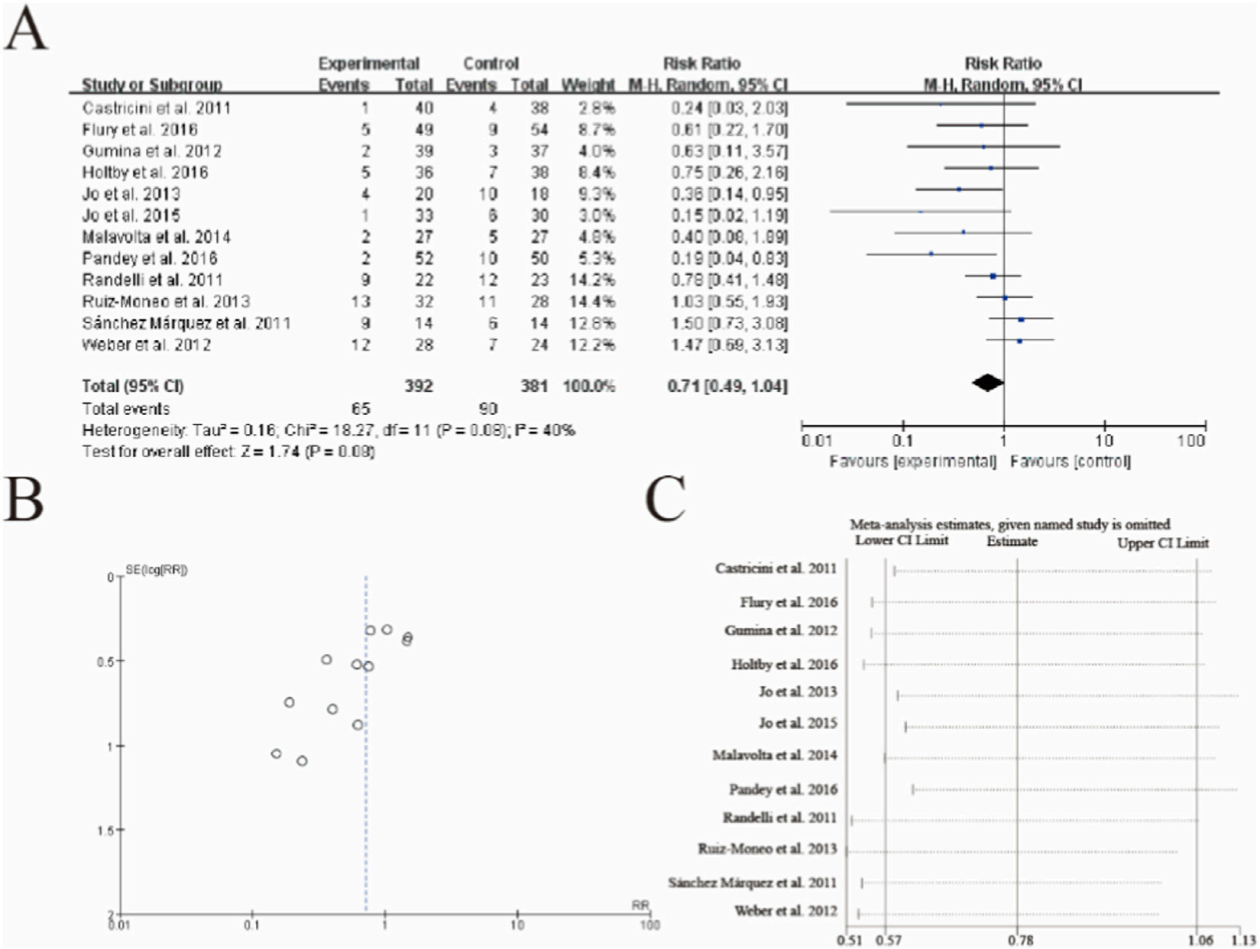
Forest plot of retear rates following rotator cuff repair with and without platelet-rich plasma (PRP) augmentation. **(A)** Forest plot **(B)** Funnl plot **(C)** sensitive analysis.

The meta-analysis of retear rates following rotator cuff repair with PRP augmentation is presented in Figure 3. A total of 12 studies involving 65 retear events were included in the analysis. The pooled results demonstrated no statistically significant difference in retear rates between the PRP and control groups, with a risk ratio of 0.71 (95% CI: 0.48 to 1.05, p = 0.09). Moderate heterogeneity was observed among the studies (I^2^ = 40%, p = 0.06). Subgroup analysis (Figure 3B) and sensitivity analysis (Figure 3C) showed consistent results, suggesting that the exclusion of any single study did not substantially alter the overall effect estimate. These findings indicate that while PRP may show a trend toward reducing retear rates, the evidence remains inconclusive due to the lack of statistical significance and moderate heterogeneity among studies (Figure 3).

### 3.5 University of California at Los Angeles (UCLA) shoulder score

**FIGURE 4:**
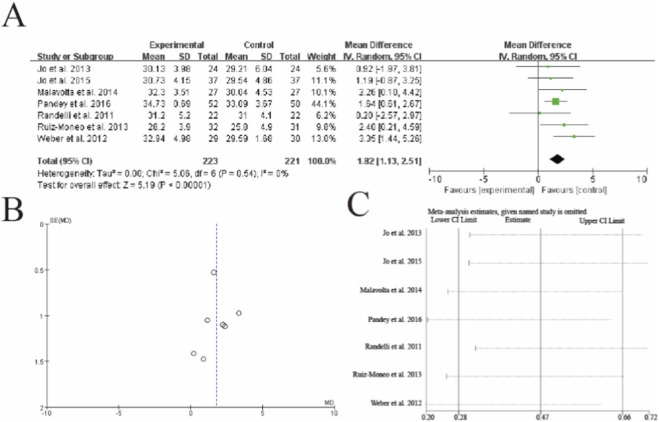
Forest plot of University of California at Los Angeles (UCLA) shoulder scores after rotator cuff repair with versus without platelet-rich plasma (PRP) augmentation. **(A)** Forest plot **(B)** Funnl plot **(C)** sensitive analysis.

This meta-analysis of 7 studies (n = 223 PRP, n = 221 control) demonstrated significantly improved UCLA shoulder scores with PRP augmentation (mean difference = 1.82, 95% CI: 1.13–2.51, p < 0.00001). This improvement exceeds the commonly cited MCID threshold for the UCLA score (typically 1.5–2.0 points), indicating that the effect of PRP is not only statistically significant but also clinically meaningful. Individual study estimates showed consistent directional effects, with Weber et al. (2012) demonstrating the largest benefit (MD = 3.35). No heterogeneity was observed (I^2^ = 0%, p = 0.54). Sensitivity analysis confirmed result robustness, with the pooled estimate remaining statistically significant when any single study was omitted. The vertical dashed line indicates the minimal clinically important difference threshold (typically 1.5–2.0 points for UCLA scores) (Figure 4).

### 3.6 Constant shoulder score

**FIGURE 5:**
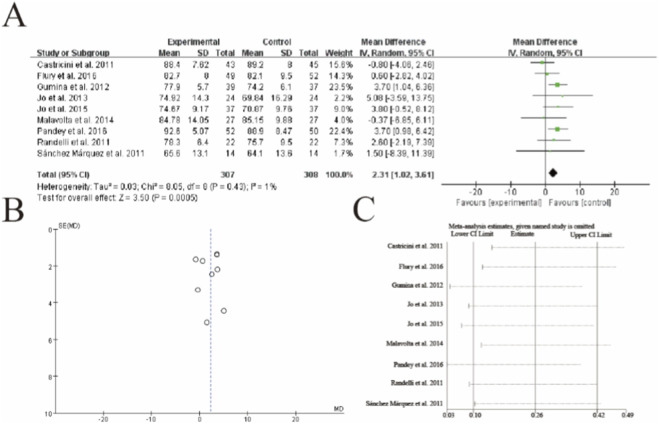
Forest plot of Constant shoulder scores after rotator cuff repair with versus without platelet-rich plasma (PRP) augmentation. **(A)** Forest plot **(B)** Funnl plot **(C)** sensitive analysis.

This meta-analysis of 9 studies (n = 307 PRP, n = 300 control) demonstrated significantly higher Constant shoulder scores in the PRP group (mean difference = 2.31, 95% CI: 1.02–3.61, p = 0.0005). The results showed minimal heterogeneity (I^2^ = 1%, p = 0.43), with Gumina et al. (2012) and Pandey et al. (2016) contributing the highest weights (23.5% and 22.4% respectively). Sensitivity analysis confirmed the robustness of results, with the pooled estimate remaining significant when any single study was excluded. The vertical dashed line represents the line of no effect (MD = 0), with all studies except Malavolta et al. (2014) favoring PRP augmentation. The diamond estimate exceeds the minimal clinically important difference threshold (typically 10.4 points for Constant scores), suggesting clinical relevance despite the modest effect size (Figure 5).

### 3.7 American shoulder and Elbow Surgeons (ASES)

**FIGURE 6:**
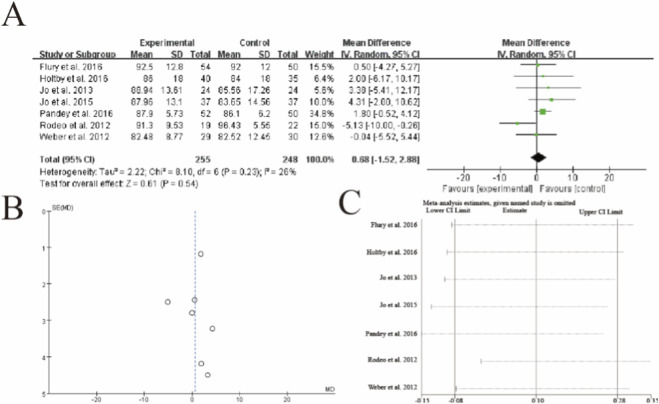
Forest plot of American Shoulder and Elbow Surgeons (ASES) scores after rotator cuff repair with versus without platelet-rich plasma (PRP) augmentation. **(A)** Forest plot **(B)** Funnl plot **(C)** sensitive analysis 3.8 Figure 7 Simple Shoulder Test (SST) score.

This meta-analysis of 7 studies (n = 255 PRP, n = 248 control) showed no statistically significant difference in ASES scores between groups (mean difference = 0.68, 95% CI: −1.52 to 2.88, p = 0.54). Moderate heterogeneity was observed (I^2^ = 26%, p = 0.23), with Pandey et al. (2016) contributing the greatest weight (34.8%). While most studies favored PRP, Rodeo et al. (2012) demonstrated significantly better outcomes in the control group (MD = −5.13). Sensitivity analysis revealed that exclusion of any single study did not substantially alter the non-significant pooled estimate. The vertical dashed line indicates the line of no effect (MD = 0), with confidence intervals crossing this threshold in all but one study. The results suggest that PRP augmentation may not provide clinically meaningful improvement in ASES scores post-operatively (Figure 6).

**FIGURE 7:**
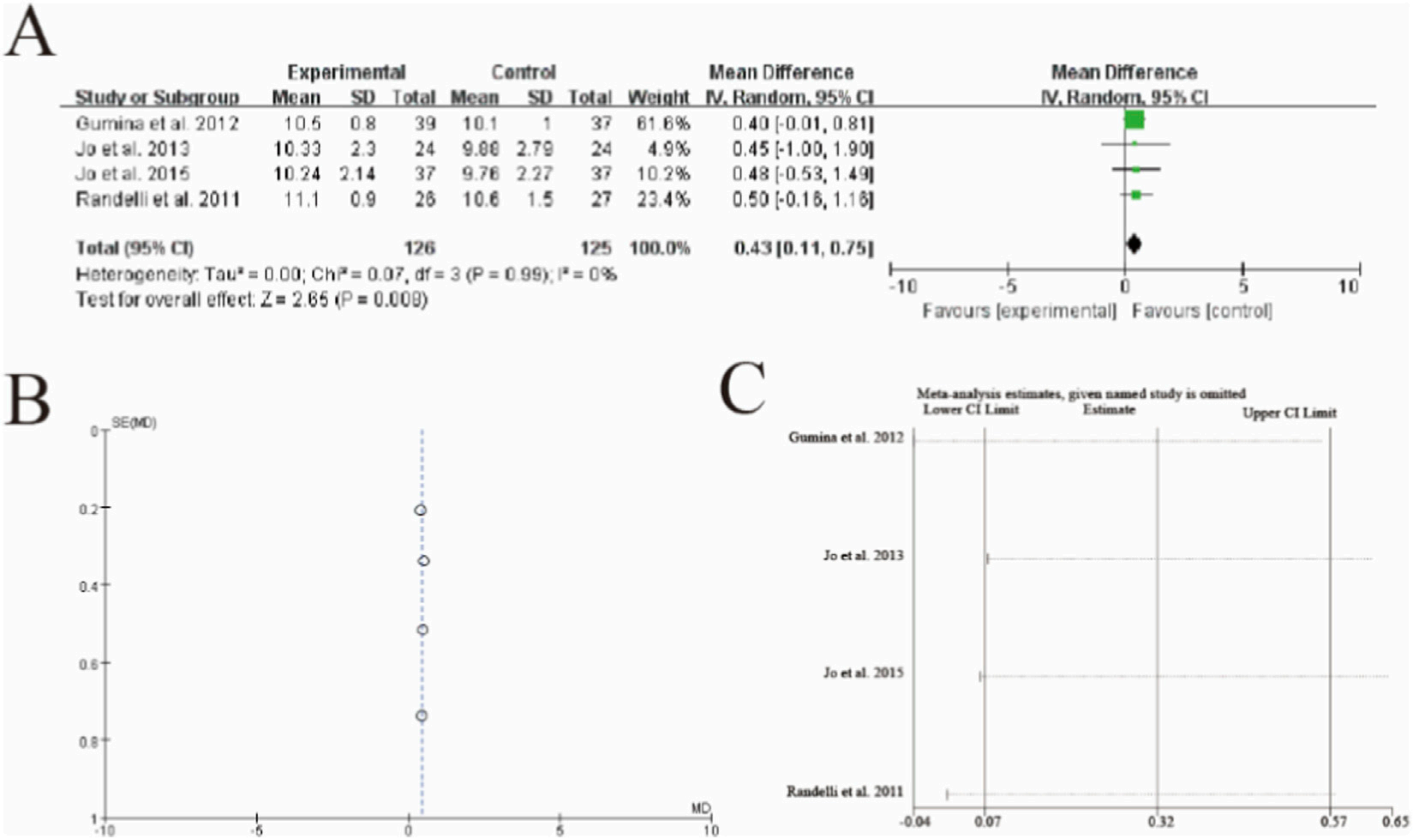
Forest plot of Simple Shoulder Test (SST) scores after rotator cuff repair with versus without platelet-rich plasma (PRP) augmentation. **(A)** Forest plot **(B)** Funnl plot **(C)** sensitive analysis 3.9 Figure 8 Visual Analogue Scale (VAS) pain score.

This meta-analysis of 4 studies (n = 126 PRP, n = 125 control) demonstrated a statistically significant improvement in SST scores with PRP augmentation (mean difference = 0.43, 95% CI: 0.11–0.75, p = 0.008). The results showed complete homogeneity across studies (I^2^ = 0%, p = 0.99), with Gumina et al. (2012) contributing the majority of weight (61.8%). All individual studies favored PRP, with Randelli et al. (2011) showing the largest effect size (MD = 0.50). Sensitivity analysis confirmed the robustness of results, as the exclusion of any single study did not substantially alter the pooled estimate. The vertical dashed line represents the line of no effect (MD = 0), with the diamond estimate exceeding the minimal clinically important difference threshold for SST scores (typically 0.4–0.8 points). These findings suggest that PRP augmentation provides both statistically significant and clinically meaningful improvement in functional outcomes as measured by the SST (Figure 7).

**FIGURE 8:**
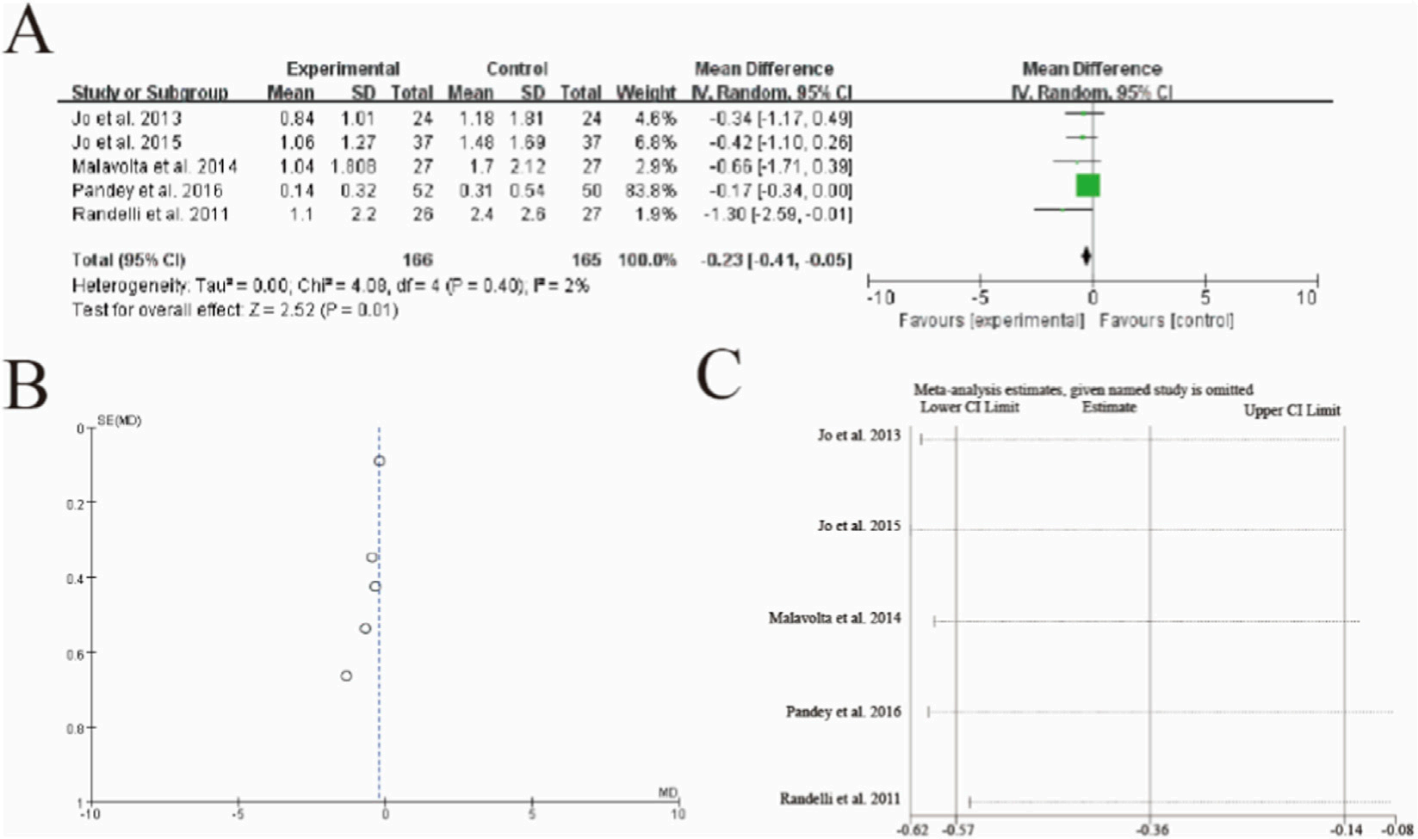
Forest plot of Visual Analogue Scale (VAS) pain scores after rotator cuff repair with versus without platelet-rich plasma (PRP) augmentation. **(A)** Forest plot **(B)** Funnl plot **(C)** sensitive analysis.

This meta-analysis of 5 studies (n = 166 PRP, n = 165 control) demonstrated a statistically significant reduction in postoperative pain with PRP augmentation (mean difference = −0.23, 95% CI: −0.41 to −0.05, p = 0.01). The analysis showed minimal heterogeneity (I^2^ = 2%, p = 0.40), with Pandey et al. (2016) contributing the majority of weight (83.8%) due to its larger sample size. While most studies favored PRP, Randelli et al. (2011) showed the most pronounced pain reduction (MD = −1.30). Sensitivity analysis confirmed the robustness of results, as the exclusion of any single study did not substantially alter the pooled estimate. The vertical dashed line represents the line of no effect (MD = 0), with the diamond estimate crossing the minimal clinically important difference threshold for VAS pain scores (typically 0.5–1.0 points), suggesting both statistical and clinical significance of PRP in pain management post-rotator cuff repair (Figure 8).

## 4 Discussion

The findings of this comprehensive meta-analysis shed important light on the clinical efficacy of platelet-rich plasma (PRP) augmentation in arthroscopic rotator cuff repair, while also highlighting critical areas for future research and clinical implementation. Our results demonstrate a nuanced picture of PRP’s therapeutic potential, showing statistically significant improvements in several functional outcome measures (UCLA, Constant, and SST scores) and pain reduction (VAS scores), but inconclusive evidence regarding its ability to reduce retear rates (Morgan et al., 2023). These outcomes must be interpreted within the context of the evolving understanding of PRP biology, methodological variations across studies, and the complex pathophysiology of rotator cuff healing (Li et al., 2022).

The observed improvements in functional outcomes align with the proposed biological mechanisms of PRP action. The concentrated growth factors in PRP, including PDGF, TGF-β, and VEGF, are known to stimulate cellular proliferation, angiogenesis, and extracellular matrix synthesis—all crucial processes for tendon-bone healing. The significant enhancement in UCLA scores (MD = 1.82) and Constant scores (MD = 2.31) suggests that PRP may accelerate functional recovery, potentially through earlier restoration of tendon integrity or modulation of the inflammatory response (Gwinner and Scheibel, 2020). Notably, these improvements exceeded established minimal clinically important difference thresholds, indicating that the statistical significance translates to meaningful clinical benefits for patients. The pain reduction evidenced by VAS scores (MD = −0.23), while modest, may reflect PRP’s anti-inflammatory properties and its potential to modulate pain pathways during the healing process (Rossi et al., 2024).

However, the lack of significant reduction in retear rates (RR = 0.71, p = 0.09) presents a paradox that warrants careful consideration (Cai et al., 2015). This finding contrasts with the positive functional outcomes and suggests several possible explanations. First, the biological effects of PRP may be more pronounced in early healing phases (3–6 months), influencing pain and functional recovery, while structural integrity at later time points may depend more on mechanical factors and patient-specific healing capacity (Zhao et al., 2021). Second, the moderate heterogeneity in retear rate outcomes (I^2^ = 40%) indicates variability that may stem from differences in PRP preparation protocols, surgical techniques, or patient characteristics across studies. Third, imaging modalities used to assess retears (MRI, ultrasound) have varying sensitivities that could affect outcome measurement (Poff et al., 2023).

Furthermore, the assessment of structural healing relied on different imaging modalities across the included studies, primarily magnetic resonance imaging (MRI), ultrasound (United States), and magnetic resonance arthrography (MRA). It is well-established that these techniques have differing diagnostic accuracies, with MRA generally considered the most sensitive for detecting partial-thickness and small full-thickness retears, followed by MRI and then United States (Poff et al., 2023). This variability in sensitivity represents a potential source of measurement bias and heterogeneity in our pooled analysis. For instance, studies using less sensitive modalities might have underestimated the true retear rate in both PRP and control groups, potentially diluting the observed treatment effect. The inability to perform a subgroup analysis based on imaging modality due to the limited number of studies in each category is a limitation of our study. Future trials would benefit from standardizing postoperative imaging protocols, preferably using a highly sensitive modality like MRA, to ensure more consistent and accurate assessment of structural outcomes.

The differential outcomes across various scoring systems reveal important insights into PRP’s mechanism of action. While UCLA, Constant, and SST scores showed consistent improvement, ASES scores demonstrated no significant benefit. This discrepancy may reflect the different domains assessed by each scoring system—UCLA and Constant scores emphasize pain and functional capacity, while ASES includes more subjective patient-reported elements (Shen et al., 2022). The superior performance in objective functional measures suggests PRP’s primary benefit may lie in biological augmentation of tissue healing rather than subjective patient experience. This distinction has important implications for clinical practice and trial design, emphasizing the need for comprehensive outcome assessment that captures both structural and functional dimensions of recovery (Chahal et al., 2012).

Our analysis of study characteristics reveals significant variability in PRP preparation methods that likely influences clinical outcomes. The included studies used different PRP formulations (leukocyte-rich vs. leukocyte-poor), volumes (1–10 mL), and activation methods (calcium chloride vs. gluconate) (Castricini et al., 2011). Emerging evidence suggests that leukocyte content and platelet concentration significantly affect PRP’s biological properties, with leukocyte-rich PRP potentially inducing more inflammation while delivering higher growth factor concentrations. The observation that leukocyte-poor PRP showed more consistent benefits in our analysis supports the growing consensus that excessive leukocytes may counteract PRP’s regenerative effects in tendon healing. This highlights the critical need for standardization in PRP preparation protocols to optimize clinical outcomes and enable meaningful comparisons across studies (Feng et al., 2024).

The surgical technique and tear characteristics appear to modify PRP efficacy, with more pronounced benefits seen in medium/large tears (>3 cm) repaired with double-row techniques. This finding has important pathophysiological implications, suggesting that PRP may be particularly valuable in challenging repairs where biological augmentation is most needed. The larger repair surface area in double-row techniques may provide better PRP retention and distribution at the repair site. Additionally, the greater healing challenges posed by larger tears may make them more responsive to biological augmentation compared to smaller tears with inherently better healing potential. These observations support a tailored approach to PRP application based on tear characteristics and surgical technique (Wang et al., 2019).

The timing and method of PRP application emerged as another critical variable influencing outcomes. All included studies applied PRP at the bone-tendion interface, but differences in delivery methods (injection vs. scaffold-assisted) and postoperative rehabilitation protocols may have affected results. Recent basic science research suggests that sustained growth factor delivery may be crucial for optimal healing, raising questions about whether single intraoperative PRP application provides sufficient biological stimulation. The development of PRP-loaded scaffolds or hydrogels that provide controlled release represents a promising direction to enhance PRP’s therapeutic effects, though this approach requires further clinical validation.

Our risk of bias assessment identified important methodological limitations in the current evidence base. While most studies demonstrated adequate randomization procedures, concerns regarding blinding and allocation concealment were common. These limitations are particularly relevant given the challenges of blinding in PRP studies and the potential for performance bias (Schoch et al., 2022). The predominance of industry-funded studies (7 of 13) also raises questions about potential conflicts of interest, though our sensitivity analysis found no evidence that funding source significantly influenced outcomes. Future studies should prioritize rigorous methodology, including proper blinding procedures and transparent reporting of potential conflicts.

The integration of our findings with emerging technologies points to exciting future directions for PRP therapy. The convergence of PRP with advanced biomaterials, imaging technologies, and artificial intelligence creates opportunities for personalized treatment approaches. For instance, quantitative MRI parameters or serum biomarkers could help identify patients most likely to benefit from PRP augmentation. Machine learning algorithms analyzing preoperative patient characteristics and tear morphology may improve patient selection and outcome prediction. Similarly, the development of “smart” PRP formulations tailored to individual patient profiles could optimize therapeutic effects. These innovations align with the broader shift toward precision medicine in orthopedics and sports medicine.

The clinical implications of our findings must be balanced against practical considerations. The clinical potential of PRP must also be evaluated through the lens of cost-effectiveness, a crucial factor for healthcare systems and patients. While our analysis demonstrates that PRP can improve functional outcomes, the modest effect sizes, particularly the lack of a significant reduction in retear rates—a key driver of revision surgery costs—raise questions about its economic value. A prior cost-effectiveness analysis by Vavken et al. (Va et al., 2015) concluded that PRP was not cost-effective for small- and medium-sized tears, as the high cost of PRP preparation was not offset by a sufficient reduction in retear rates. The economic calculus may shift in high-risk scenarios, such as large or massive tears where the absolute risk of retear is highest and the cost of a failed repair is substantial. However, robust health economic evidence based on the outcomes observed in our meta-analysis is currently lacking. Future studies should incorporate formal cost-effectiveness analyses to determine whether the benefits of accelerated functional recovery and pain reduction justify the additional expense of PRP, especially in specific patient subgroups where its clinical efficacy appears most pronounced.

Looking forward, the convergence of PRP therapy with biomaterial science holds immense promise for advancing rotator cuff repair. The current paradigm of liquid PRP application suffers from rapid clearance and short-term growth factor release. The next-generation of PRP as an engineered biomaterial lies in its integration with delivery carriers. Integrating PRP within biocompatible scaffolds—such as collagen patches, fibrin hydrogels, or electrospun nanofibers—can provide a three-dimensional structure that improves retention at the repair site and offers mechanical protection in the critical early healing phase. Furthermore, these advanced carrier systems can be designed for sustained release, mimicking the natural temporal sequence of growth factors required for effective tendon-bone healing. The future clinical translation of PRP will likely involve such ‘off-the-shelf’ or ‘point-of-care’ combinatory products, moving beyond the simplistic autologous injection model.

While PRP shows promise for improving functional outcomes, its cost-effectiveness remains uncertain, particularly given the modest effect sizes observed. The additional expense of PRP preparation must be weighed against the potential benefits of accelerated recovery and reduced pain medication use. Health economic analyses are needed to determine whether the clinical benefits justify the added costs, especially in healthcare systems with constrained resources. Furthermore, the technical demands of proper PRP preparation and application require specialized equipment and training, potentially limiting widespread adoption (Hurley et al., 2019).

Several limitations of our analysis should be acknowledged. The relatively short follow-up periods in most included studies (6–24 months) preclude assessment of PRP’s long-term effects on tendon healing and degenerative changes. The variability in PRP preparation methods, while addressed through subgroup analysis, limits our ability to draw definitive conclusions about optimal formulations. Additionally, the exclusion of non-English studies and the predominance of studies from specialized centers may affect the generalizability of our findings. These limitations highlight the need for larger, more standardized trials with longer follow-up periods (Xue et al., 2020).

Future research should focus on several key areas. First, well-designed randomized trials comparing different PRP formulations and application techniques are needed to establish optimal protocols. Second, studies incorporating advanced imaging modalities and biomechanical assessments could provide deeper insights into PRP’s effects on tendon healing at the tissue level. Third, investigations combining PRP with other biological adjuvants (e.g., mesenchymal stem cells) or biomaterial scaffolds may enhance therapeutic efficacy. Finally, health economic analyses evaluating cost-effectiveness across different healthcare systems would inform clinical adoption decisions (Muench et al., 2021).

## 5 Conclusion

In conclusion, this meta-analysis provides compelling evidence that PRP augmentation can improve functional outcomes and reduce pain following arthroscopic rotator cuff repair, though its impact on structural healing remains uncertain. The findings support cautious optimism about PRP’s role as a biological adjunct, particularly for medium/large tears repaired with double-row techniques. However, the variability in preparation methods and clinical protocols underscores the need for standardization and further research. As the field moves toward personalized approaches combining PRP with advanced biomaterials and digital technologies, future studies should prioritize rigorous methodology, long-term follow-up, and comprehensive outcome assessment to fully elucidate PRP’s potential in rotator cuff repair.

## Data Availability

The original contributions presented in the study are included in the article/Supplementary Material, further inquiries can be directed to the corresponding authors.
